# Determinants of male fertility in the Western Pacific Region: environmental, biological, and lifestyle influences

**DOI:** 10.1016/j.lanwpc.2025.101716

**Published:** 2025-12-08

**Authors:** David A. Skerrett-Byrne, Lee M. Ashton, Brett Nixon, Philip J. Morgan

**Affiliations:** a Institute of Experimental Genetics, Helmholtz Zentrum München, German Research Center for Environmental Health Neuherberg, Germany; b German Center for Diabetes Research (DZD) Neuherberg, Germany; c Infertility and Reproduction Research Program, Hunter Medical Research Institute, New Lambton Heights, NSW, Australia; d School of Biomedical Sciences and Pharmacy, College of Health, Medicine and Wellbeing, University of Newcastle, Awabakal Country, Callaghan, NSW, Australia; e Centre for Active Living and Learning, College of Human and Social Futures, School of Education, University of Newcastle, Awabakal Country, Callaghan, NSW, Australia; f Active Living and Learning Research Program, Hunter Medical Research Institute, New Lambton Heights, NSW, Australia; g Research Centre for Reproductive Sciences, School of Environmental and Life Sciences, College of Engineering, Science and Environment, University of Newcastle, Awabakal Country, Callaghan, NSW, Australia

**Keywords:** Fertility, Paternal preconception health, Male infertility, Sperm, Interventions, Epigenetics, Sperm proteomics, Environmental factors, Transgenerational health, Assisted reproductive technologies, Physical activity, Mental health, Men's health, Nutrition, Weight management, Substance use

## Abstract

Over the past half-century, global fertility rates have declined, with the Western Pacific Region (WPR) experiencing a particularly notable drop. A recent World Health Organisation-commissioned report identified the WPR as exhibiting the highest infertility prevalence at 23.2%, compared to the global average of 17.5%. While the drivers of this decline are complex, one key contributor is male infertility, yet it remains under addressed in research and policy. In this paper, we synthesise current evidence on male infertility with a focus on the WPR. Specifically, we explore environmental, biological, and demographic correlates of male infertility, examine molecular mechanisms regulating sperm function and assess the impact of lifestyle interventions. Our findings highlight significant gaps in regional evidence, advocating for targeted research and culturally tailored interventions to enhance preconception male health within the WPR. Based on this synthesis, we propose preventive strategies and evidence-based recommendations to improve male preconception health in the region.

## Graphical abstract

**Figure:**
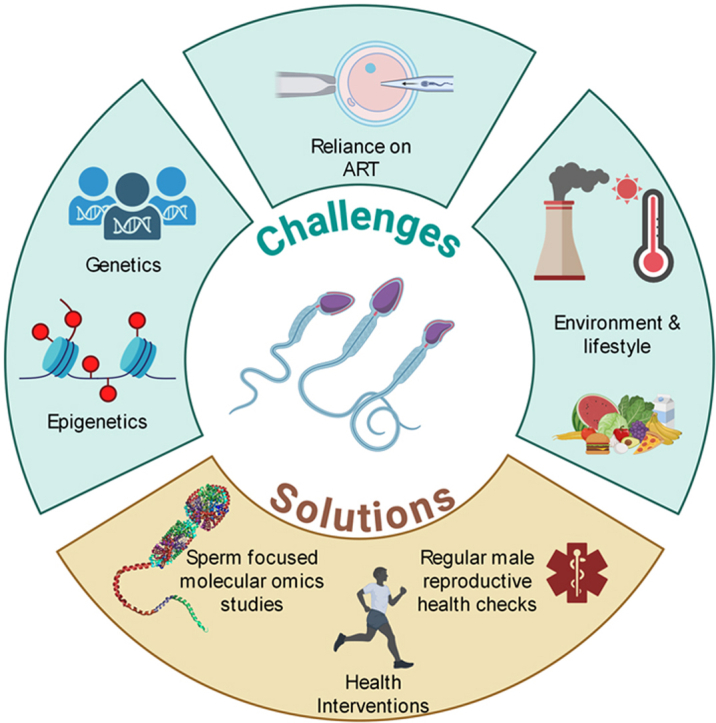

## Introduction

**Fig. 1:**
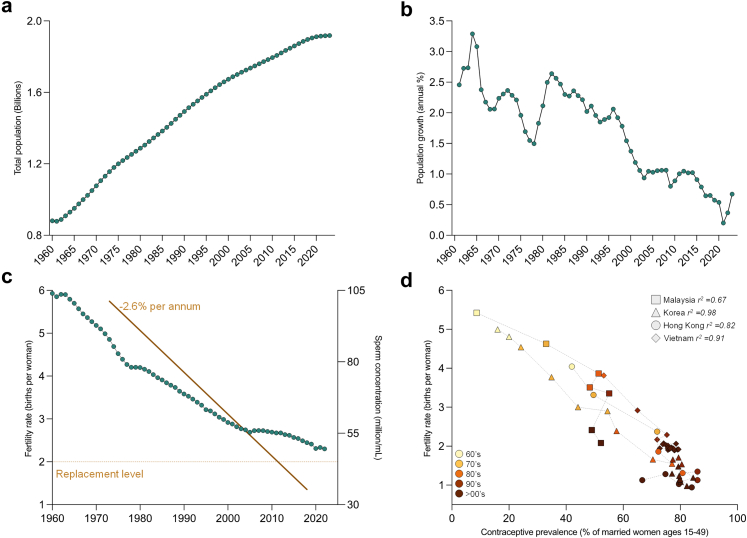
Overview of the population and fertility rates in the Western Pacific Region (WPR). Data were sourced from the World Bank Group from the WPR nations, graphs display (a) the total population (in billions) and (b) population growth rate (annual %). (c) Fertile rate (births per women) from WPR are graphed over time and the second y-axis incorporates findings from Levine et al.,^3^ on global sperm concentrations (millions/mL) from 1973 to 2018. (d) Correlation plot of fertility rate against the prevalence (accessibility) of contraceptives, across decades of four WPR nations (source: World Bank, 2025).^2^

Over the past half-century, global fertility rates have experienced a marked decline, an undercurrent masked by the growing global population attributed to demographic momentum (i.e., population growth continues despite families having fewer children).^1^ This trend is exemplified within the Western Pacific Region (WPR), a demographically diverse and rapidly evolving landscape, where advanced economies such as Japan, Australia, and China are experiencing some of the world's lowest fertility rates, while others in Southeast Asia and the Pacific Islands (e.g., Cambodia and Papua New Guinea) maintain higher, yet declining, total fertility rates (TFR).^2^ Collectively the population of the WPR has surged from approximately 0.8 to nearly 2 billion over the past six decades (Fig. 1a),^2^ despite a progressive decline in the annual population growth to around 0.5% (Fig. 1b).^2^ This trend is reflected in a parallel decline in the average number of births by a woman, from 5.9 to 2.3 over the same time period (Fig. 1c); a TFR that is fast approaching the replacement levels of 2.1 children needed to maintain a stable population.^1^

While the drivers of such fertility decline are undeniably complex, one of the key contributors is male infertility, a pathology that is responsible for approximately half of all cases of couple infertility.^4^^,^^5^ Indeed, defective sperm function ranks as the largest single, defined cause of human infertility.^6^ Although this key public health concern is gradually gaining the attention it merits, it still represents an under addressed pathology in both research and policy domains. Simultaneously, societal and behavioural changes, particularly the increased adoption of contraceptives, have contributed to declining fertility rates across several WPR nations (Fig. 1d).^2^ These patterns reflect delayed family planning, with couples increasingly postponing childbearing into later reproductive years. This trend is relevant to male fertility, as advancing paternal age is associated with reduced sperm quality, increased DNA fragmentation, and a higher risk of adverse offspring outcomes.^7^^,^^8^ Parallel to these demographic shifts, seminal work by Levine et al.^3^^,^^9^ has documented a concerning acceleration of global declines in sperm production in recent decades. It follows that the concentration of sperm cells within an ejaculate has fallen from 101.2 to 35.3 million/mL between 1973 and 2018; amounting to −2.64% per annum post-2000 (Fig. 1c).

Infertility, as defined by the World Health Organization (WHO), is the inability to achieve pregnancy after at least 12 months of regular unprotected intercourse and affects approximately one in six couples globally.^10^ Despite its high prevalence, the responsibility of infertility is disproportionately placed on women, who bear the majority of the health, emotional and financial burdens.^11^^,^^12^ This gendered imbalance is sharply illustrated by Sharp et al.,^13^^,^^14^ who highlight that within the field of developmental origins of health and disease (DOHaD), maternal health is referenced nearly 20 times more often than paternal health in the scientific literature. This is partly rooted in entrenched cultural norms surrounding masculinity, which frame help-seeking and vulnerability as signs of weakness.^15^^,^^16^ This imbalance has profound implications, for not only does it limit scientific understanding of paternal contributions, but it also shapes clinical priorities and funding allocations. Recent studies have begun to dismantle this imbalance, now implicating paternal lifestyle behaviours, such as smoking, poor diet, heat exposure, physical activity and obesity, in altering the epigenetic cargo of sperm. These lifestyle behaviours not only have the potential to negatively impact a male's fertility status but also exert influence over early embryonic development and offspring health^17^, ^18^, ^19^, ^20^, ^21^, ^22^; alluding to a form of male-mediated transgenerational inheritance. Such insights necessitate a re-evaluation of paternal health not merely as a contributor to conception, but as an active determinant of child health outcomes.

Male infertility is not a singular disease but rather a multifactorial syndrome encompassing genetic, environmental, behavioural, and socio-demographic components. Genetically, at least 120 gene-disease relationships have been validated for male infertility,^23^ yet 60–70% of cases remain idiopathic (defined as failure to conceive after standard fertility evaluation has excluded identifiable genetic, hormonal or anatomical causes^24^) due to limitations in clinical genetic screening. In fact, despite these advances, only ∼4% of cases currently have an established genetic cause,^25^ highlighting a substantial knowledge gap of what combination of factors are driving infertility in these 60–70% of idiopathic cases? Environmental factors also play a significant role, with exposure to persistent organic pollutants, endocrine-disrupting chemicals (e.g., PFAS, BPA), air pollution, and agricultural pesticides now prevalent in daily life, and notably elevated in certain WPR subregions, all correlated with male reproductive pathologies.^26^, ^27^, ^28^ Compelling epidemiological and pre-clinical evidence has increasingly linked these exposures to reduced sperm quality and testicular dysgenesis.^29^, ^30^, ^31^ Concurrently, shifts in increasing age at which men choose to father children, alongside lifestyle factors, such as dietary shifts, rising obesity rates, smoking, and inactivity, compound these risks, particularly in densely urbanised areas.^32^, ^33^, ^34^, ^35^, ^36^

This review synthesises emerging trends in male infertility with a regional focus on the Western Pacific. Specifically, we examine environmental, biological, and demographic correlates of male infertility, explore molecular mechanisms regulating sperm function, evaluate the effectiveness of lifestyle behaviour interventions, and provide a roadmap of preventive strategies and evidence-based recommendations for improving male preconception health.

## Methods

### Search strategy

We conducted a comprehensive review of the existing literature to identify key evidence relating to male infertility, particularly in the context of lifestyle and environmental factors influencing sperm quality and fertility outcomes. Databases searched included PubMed and Google Scholar, covering studies published from 2000 until May 2025. The search was not limited to English language publications, thereby capturing regional and international evidence within the WPR nations. Despite this, the use of English-language search terms means that studies published exclusively in other languages may not have been captured. Search terms included combinations of “male infertility”, “sperm parameters”, “preconception health”, “lifestyle behaviours”, “diet”, “physical activity”, “psychological stress”, “environmental factors”, “endocrine-disrupting chemicals”, and “Western Pacific Region”. Relevant studies included randomised controlled trials (RCTs), systematic and scoping reviews, meta-analyses, cohort studies, and seminal narrative reviews. Studies were excluded if they did not specifically provide explicit clinically meaningful outcomes such as sperm parameters, pregnancy, or live birth rates.

## Correlates and drivers of male infertility

### Male infertility phenotypes

A recent WHO commissioned report highlighted the WPR as exhibiting the highest infertility prevalence at 23.2%, compared to the global average of 17.5%.^10^ Clinically, male infertility phenotypes are largely defined through descriptive diagnoses based on semen analysis, encompassing azoospermia (complete absence of sperm), oligospermia (low sperm count), asthenozoospermia (poor sperm motility), and teratozoospermia (abnormal sperm morphology). Frequently, patients present with combined phenotypes, such as oligoasthenoteratozoospermia (OAT syndrome), reflecting multifaceted disruptions in sperm quality and quantity.^25^ Notably, the quality of human sperm cells is generally considered to be much lower than that of most other mammalian species. Indeed, normozoospermic ejaculates from healthy donors are characterised by as few as ∼4% sperm that display normal morphology, 42% motility and ∼54% viability.^37^^,^^38^ Additionally, hormonal imbalances, particularly involving testosterone deficiency and dysregulation of follicle-stimulating hormone (FSH) and luteinising hormone (LH), also represent crucial drivers of male infertility.^25^ Elevated FSH levels commonly indicate impaired spermatogenesis, whereas low testosterone may signify broader hypogonadism, reflecting deeper endocrine dysfunction. However, despite significant advances in our understanding of male infertility, >70% of cases remain classified as idiopathic or unexplained.^25^ In fact, only about 4% of cases have an established genetic link,^25^ although recent concerted efforts have identified over 100 high-probability genes associated with male infertility.^23^ This raises the question of what combination of factors are driving infertility in the remaining 70% of cases? (See section: Mechanisms/mediators)

Increasing rates of global infertility are fuelling unprecedented demand for couples to circumvent these issues through Assisted Reproductive Technologies (ART) such as *in vitro* fertilisation (IVF) and intracytoplasmic sperm injection (ICSI). Over the past 15 years the WPR has witnessed a marked increase in ART uptake, with numbers of ART cycles nearly doubling in Japan,^39^ Australia and New Zealand.^40^^,^^41^ In Australia and New Zealand, nearly two-thirds of all cycles of ART are now ICSI, regardless of underlying infertility issue.^42^ This ever increasing uptake of ART has given rise to the concept of an “ART paradox” or the “infertility trap”,^43^ whereby diminished evolutionary selection pressures may perpetuate infertility-linked genetic variants within the population, building an inevitable dependency in subsequent generations.^1^^,^^44^ These complexities combine to present a compelling case for additional research into the largely unknown aetiologies underlying male infertility.^45^

### Behavioural and lifestyle factors

The impact of behavioural and lifestyle factors (i.e., diet, physical activity, sleep, substance use, and psychological stress) on male fertility has received increasing recognition, particularly given the significant proportion of unexplained infertility cases.

Unhealthy dietary patterns characterised by high consumption of processed foods, sugars, saturated fats, and low nutrient density are frequently associated with reduced semen quality and hormonal disruption.^46^, ^47^, ^48^ Conversely, traditional dietary practices rich in whole foods, fibre, antioxidants, and micronutrients typically support improved spermatogenesis and hormonal balance.^46^^,^^49^

The WPR encompasses diverse cultures, religions, political systems, socioeconomic contexts, lifestyles, and environmental factors, resulting in greater variation in dietary patterns and other lifestyle behaviours compared with other regions.^50^ Over the past 30 years, dietary intakes in the WPR have shifted considerably. Positive changes include increased consumption of oils, fruits and vegetables, alongside reductions in sodium, saturated fats and trans fats.^50^ These trends align with several features of the Mediterranean diet, one of the most extensively studied dietary patterns, which is associated with a range of health benefits,^51^ including improved semen quality.^52^ At the same time, however, there has been an increase in the consumption of foods with detrimental effects on semen quality,^46^ such as processed foods and sugar-sweetened beverages.^50^

Obesity, increasingly prevalent worldwide, is notably detrimental to male fertility, impairing sperm parameters,^53^ inducing hormonal imbalances (e.g., testosterone and oestrogen levels),^54^ and contributing to systemic inflammation and oxidative stress.^55^ A contributing factor to obesity is low physical activity, with activity levels in WPR having declined over the past 30 years,^50^ which mirrors global trends.^56^ In contrast those who maintain a physically active lifestyle exhibit improved sperm quality including increased count, motility, and morphology, as well as favourable hormonal levels (increased testosterone, FSH and LH) compared to those of a sedentary nature.^57^^,^^58^ However, prolonged or excessive exercise may mitigate these positive benefits reflected in hormonal imbalances.^58^

Other lifestyle factors such as alcohol and tobacco consumption are widely recognised as detrimental to male reproductive health. Smoking is robustly associated with decreased sperm concentration, motility, and morphology, as well as impacting hormones.^59^^,^^60^ Interestingly, recent work with infertile men who cease to smoke for 3 months observed a marked improvement across these sperm parameters,^61^ although further studies are required. Alcohol consumption, particularly in excess, is associated with increased testosterone levels,^62^ reduction in sperm concentration and morphologically normal spermatozoa.^63^ Over the past 30 years, alcohol use has increased while smoking has declined across the WPR. Nevertheless, both smoking rates and alcohol consumption in the WPR remain higher than the global average.^50^

Sleep is an often overlooked, yet critical lifestyle factor influencing male fertility. Sleep deprivation has been associated with reduced sperm concentration and motility,^64^^,^^65^ along with increased markers of oxidative stress.^65^ Given rising rates of sleep disturbance and shift work in urbanised WPR populations,^66^ further investigation into sleep quality as a modifiable determinant of reproductive health is warranted.

Lastly, an increasingly recognised factor in reproductive success is psychological stress.^67^ Psychological stress prevalence is rising globally and disproportionally impacts younger people.^68^ Chronic stress negatively affects male fertility via the hypothalamic-pituitary-gonadal (HPG) axis, notably through increased release of gonadotropin inhibitory hormone (GnIH). GnIH suppresses gonadotropin-releasing hormone (GnRH), reducing LH, FSH, and testosterone production, impairing spermatogenesis and sperm quality.^69^ With rapid urbanisation in WPR, elevated stress levels are observed,^70^^,^^71^ stressing the need for targeted interventions focusing on psychological well-being as an essential component of holistic reproductive healthcare strategies.

### Environmental factors

Our ever-changing world influences our reproductive health, with exposure to pollutants, occupational hazards, and broader climatic changes emerging as critical considerations for male infertility across the WPR.^72^, ^73^, ^74^, ^75^

Exposure to endocrine-disrupting chemicals (EDCs), including per- and poly-fluoroalkyl substances (PFAS), bisphenol A (BPA), phthalates, pesticides, and heavy metals, is increasingly recognised for their deleterious effects on male reproductive function.^30^ EDCs interfere with sex hormones (notably testosterone),^76^^,^^77^ impairing spermatogenesis through abnormal sperm morphology,^78^ sperm DNA damage^79^ and motility.^78^^,^^80^ The WHO/UNEP 2017 report on endocrine-disrupting chemicals has highlighted the growing international concern about EDCs, reinforcing the urgent need for improved regulatory interventions, frameworks and public health policies aimed at reducing exposure to these substances.^81^ The report highlights policy initiatives in several WPR nations including Japan, South Korea, and Australia. South Korea's K-REACH, Regulation on the Registration and Evaluation of Chemical, requires registration and evaluation of chemical substances, but currently does not include a specific framework for identifying or managing EDCs. In Australia, the National Industrial Chemicals Notification and Assessment Scheme (NICNAS) oversees chemical risk management for all new chemicals except industrial chemicals. Additionally, the NICNAS lacks a dedicated classification system for EDCs and relies primarily on international assessments. As noted in the WHO/UNEP report, these frameworks often lack clear criteria for EDC identification, rely on external data, or remain at early stages of development, pointing to the need for more robust, harmonised approaches in the WPR.^81^

Occupational factors, particularly prevalent in certain industries within the WPR, significantly impact male fertility. Heat stress, common in industrial, mining, and agricultural work, negatively affects spermatogenesis by elevating testicular temperatures, thereby compromising sperm quality and production.^82^, ^83^, ^84^ Exposure to industrial chemicals, radiation and even electromagnetic fields further compounds fertility risks, with hormonal disruption, increased sperm DNA damage and decreased sperm motility.^85^, ^86^, ^87^, ^88^ Occupational health policies must prioritise protective measures and exposure mitigation strategies to safeguard reproductive health.

Lastly, climate change introduces additional stressors to male fertility through rising environmental temperatures and increased frequency of heatwaves,^89^^,^^90^ potentially exacerbating scrotal heat stress and negatively influencing sperm quality and epigenetic cargo.^17^^,^^91^^,^^92^ Preliminary evidence indicates a concerning correlation between rising temperatures and declining semen parameters,^93^^,^^94^ suggesting that global warming may increasingly pose significant reproductive risks. Understanding these climate-related impacts and developing adaptive strategies are critical in managing future reproductive health outcomes.

The intersection of behavioural, lifestyle, and environmental factors underscores the complexity of addressing male infertility comprehensively. Public health strategies that integrate dietary guidance, physical activity promotion, mental health support, substance misuse prevention, and environmental protection will be crucial in addressing the multifaceted nature of infertility in the WPR.

### Socio-demographic factors

Socio-demographic factors play an increasingly important role in shaping male fertility outcomes across the WPR, where economic development, urbanisation, and shifting cultural norms are altering reproductive behaviours.

Paternal age is a well-recognised factor influencing male fertility. As men age, sperm parameters, including volume, motility, and morphology, tend to decline.^7^ Advanced paternal age is also associated with increased rates of de novo mutations, DNA fragmentation, and epigenetic alterations in sperm.^8^ These changes have been linked to changes in the sperm epigenome^95^ and a higher risk of adverse reproductive outcomes, including reduced embryo quality,^96^ miscarriage,^97^ and increased risk of cancer and neuropsychiatric disorders in offspring.^98^^,^^99^

Economic and cultural influences further shape fertility trends.^43^ Globally and in many WPR countries, rising costs of living and prolonged education or career development have delayed the average age of first childbearing.^43^ In settings where traditional family size preferences are still strong, these shifts may create tensions between cultural expectations and biological limitations.^100^ Access to fertility services also remains highly variable across the region. While countries such as Australia, Japan, and South Korea offer subsidised or regulated access to ART (60–70%),^101^ many Pacific Island nations and lower-income countries lack equitable access to fertility care due to cost, limited infrastructure, and sociocultural stigma.^102^^,^^103^

Together, these socio-demographic pressures may contribute to increased rates of infertility, particularly when delayed childbearing intersects with lifestyle and environmental risk factors. Addressing these challenges will require regionally tailored strategies that improve access to fertility education and services, support family planning autonomy, and acknowledge the complex interplay between age, culture, and reproductive health.

### Mechanisms/mediators

Although not specific to the Western Pacific Region, understanding the biological mechanisms underlying male fertility, from spermatogenesis to transit of the epididymis, is essential to contextualise regionally prevalent exposures and guide future research and interventions.

### Spermatogenesis

The male gamete endures a tortuous journey en route to the formation of a highly specialised spermatozoon, which is capable of engaging in production interactions with an oocyte to initiate the process of fertilisation. The maturation of the sperm cell (i.e. spermatogenesis) begins in specialised niches located within the basal compartment of the seminiferous tubules of the testes, wherein a sub-population of the resident spermatogonial stem cells (SSC) are stimulated to commence differentiation into progenitor spermatogonia.^104^ Once committed to this pathway, progenitor cells then undergo successive waves of mitotic divisions, amplifying their numbers and producing differentiating spermatogonia possessing the attributes necessary for entry into the meiotic phases of the spermatogenic cycle. Here, differentiating spermatogonia undergoes two rounds of meiotic divisions leading to the production of haploid spermatids bearing half the chromosome number and a distinct genetic profile to that of their parent cells. The spermatids so formed then commence a complex sequence of cytodifferentiation events collectively referred to as spermiogenesis. This final phase of spermatogonial differentiation encompasses sweeping changes in sperm cell morphology and internal architecture including extreme structural condensation of the paternal chromatin, the formation of an acrosomal vesicle adorning the anterior region of the sperm head, development of the flagellum and associated motility apparatus, and the shedding of excess cytoplasm along with the majority of the organelles that typify their somatic cell counterparts.^104^ One of the notable consequences of this dramatic cellular remodelling is that the mature sperm cell lacks the capacity for de novo gene transcription or protein translation. Thus, the functional maturation that ensues as spermatozoa descend through the extratesticular regions of the male reproductive tract (i.e. epididymis; discussed below) is reliant on the external milieu they encounter within this microenvironment.^105^

The passage of spermatozoa through the successive phases of spermatogenesis is tightly regulated by an inordinately complex array of molecular signals including hormones, growth factors, and transcription factors. Among the key hormones, testosterone (produced by Leydig cells) and follicle-stimulating hormone (FSH; secreted by the pituitary gland) are noteworthy for their crucial roles in promoting spermatogonial proliferation and differentiation, and stimulating Sertoli cells support of spermatogenesis, respectively.^106^ The reciprocal action of growth factors such as glial cell line-derived neurotrophic factor (GDNF) and fibroblast growth factor (FGF) is also held to be essential for SSC maintenance and differentiation. Thus, while GDNF promotes SSC self-renewal and prevents premature differentiation, FGF signalling contributes to the proliferation and differentiation of spermatogonia. Such signals work, at least in part, via the regulation of a suite of transcription factors that, in turn, coordinate the spatial and temporal expression patterns of the thousands of genes necessary for supporting spermatogenesis.^106^ Indeed, although knowledge of the processes that control human spermatogenesis lags well behind that of laboratory model species, recent studies have shown that the successive phases of human spermatogenesis are controlled by a complex network of molecular pathways involving in excess of 4000 genes.^107^ Ongoing efforts to curate the function of the multitude of resultant gene products hold the promise of delivering diagnostic criteria with which to dissect the mechanistic basis of various forms of spermatogenic compromise, male infertility and men's health more broadly.^108^ However, despite these advances, clinical application of genetic findings remains limited. As highlighted by Tüttelmann et al.,^25^ routine genetic testing, including karyotype analysis, Y-chromosome microdeletion screening, and CFTR genotyping, yields a definitive diagnosis in only ∼4% of unselected infertile men, rising to ∼20% in azoospermic cohorts. This underscores the current gap between molecular insights and clinical implementation.

Notably, studies exploiting these resources have shown that, beyond intrinsic regulatory factors, spermatogenesis is also acutely sensitive to various forms of contemporary paternal exposures including those associated with environmental, occupational, and lifestyle hazards; all of which have the potential to impact sperm quality and male fertility.^109^^,^^110^ Among the key insults capable of perturbing the fidelity of spermatogenesis are those that impede the thermoregulatory capacity of the scrotum, including the practice of wearing tight underwear and/or occupations that necessitate extended periods of seated activity, such as desk jobs or those in the transport industry.^111^ However, this situation can be further exacerbated by seasonal variations in ambient temperature, with numerous studies linking summer months with attendant reductions in sperm quality.^112^ Such findings are of particular concern in the Western Pacific Region, wherein the adverse consequences of anthropogenic-driven climate change have already begun to give rise to more extreme weather events, including longer and hotter summers and an increased prevalence and intensity of heat waves.^113^ By way of illustration, climate records provide evidence that the average number of consecutive days of heat stress has increased from 2 days per heat stress event from 1960 to 1999, to 4 days from 2000 to 2008 in regions such as southern Australia.^89^

In accounting for the adverse impacts of thermal stress on male reproduction, it is noteworthy that the male reproductive tract features numerous anatomical and physiological adaptations that permit optimal sperm production to proceed at temperatures that are generally 2–6 °C below that of core body temperature.^111^ Key among these is the descent of the testicles into a scrotum and sophisticated countercurrent blood flow networks, which creates a substantial temperature gradient between the body and testes. However, this thermoregulation system is not infallible and when even subtle excursions in scrotal temperature arise, they can elicit pronounced negative impacts on the germinal epithelium. These impacts include endocrine dysregulation, elevated levels of oxidative stress and an attendant rise in apoptosis, DNA damage, epigenetic changes, and abnormalities of spermiogenesis within the developing germline.^17^^,^^111^ These effects combine to reduce rates of sperm production, damage the integrity of the paternal genome and epigenome (see below), and sperm cell function more broadly. Together, these adverse consequences of thermal stress contribute to reduced male fertility and an increased risk of paternally derived pathologies in the offspring of exposed men.

Although the negative consequences of thermal stress are well documented, similar outcomes have also been reported in response to an ever-increasing spectrum of paternal exposures, having been correlated with negative impacts on semen quality; most often reflected in reduced sperm count and quality. Such exposures are broadly grouped under environmental (e.g. persistent organic pollutants, endocrine disruptors, PFAS, BPA, phthalates, pesticides, heavy metals), occupational (e.g. chemicals, radiation), and lifestyle hazards (e.g. diet and nutrition, substance use, sedentary behaviour, physiological stress, sleep deprivation, paternal age at conception).^114^ Beyond perturbation of hormonal levels, a common legacy of these exposures is an elevation in oxidative stress. Defined as an imbalance between the production of reactive oxygen species (ROS) and a cell's antioxidant defences, oxidative stress is recognised as an underlying aetiology in the majority (i.e., >80%) of male infertility cases diagnosed as idiopathic.^115^ Such stress can arise through diverse sources, but all lead to a synonymous pathway that drives sperm towards dysfunction. This commonly manifests in oxidative lesions of the sperm nucleic acid, lipid and protein content, which, in turn, promote collateral damage in the form of DNA fragmentation, peroxidative damage of cellular membranes, and cellular dysfunction. It follows that oxidative stress is directly responsible for an extensive range of infertile pathologies, including defective sperm motility and sperm-egg recognition, the latter of which ranks among the most common causes of clinical IVF failure.^116^ Although sperm cells are vulnerable to oxidative stress at all stages of their development, as discussed below, this issue becomes particularly acute after the cells leave the protective environment of the testes and begin their descent through the epididymis.

### Role of the epididymis

Notwithstanding the high degree of morphological specialisation attained during spermatogenesis, the sperm cells that leave the testes remain functionally incompetent, not only lacking the ability to swim in a progressive manner but also the potential to engage in the cascade of oocyte interactions that culminate in fertilisation.^105^ In the absence of de novo gene transcription or protein translation, the attainment of these key functional attributes rests with the exposure of sperm cells to the microenvironment they encounter within the lumen of the epididymis. This complex milieu is generated by the combined secretory and absorptive activity of the lining epithelial cells and comprises a diversity of inorganic ions, proteins, lipids and nucleic acids.^105^ Together, such factors drive the remodelling of the molecular composition of the sperm cell and ultimately confer both motility and fertilisation competency to these cells. There is growing appreciation that the scale of these changes, encompassing the loss, gain and post-translational modification of several thousand proteins,^117^ necessitates the existence of efficient mechanisms of macromolecular exchange between the epididymal epithelial cells and maturing sperm cells. Current research suggest that this intercellular communication nexus involves extracellular vesicles (EVs) secreted by the epididymal epithelium.^118^^,^^119^ Against this dynamic backdrop, while testicular defects remain a central focus of clinical investigation, it is increasingly evident that disruptions to epididymal signalling, particularly through EV-mediated transfer of proteins and small non-coding RNAs (sncRNAs), can impair the acquisition of sperm motility and fertilisation capacity.^118^^,^^120^^,^^121^ This insight raises the possibility that epididymal dysfunction may account for a substantial fraction of the >70% of infertility cases currently deemed idiopathic,^25^ representing a critical yet overlooked component in both the diagnosis and treatment of male reproductive failure.

Notably, beyond their rich lipid and proteomic cargo, epididymal EVs have also been implicated in the delivery of a complex array of sncRNA cargo to sperm cells.^118^ Moreover, there is compelling evidence that sncRNA payload relayed by epididymal EVs to recipient sperm is dramatically altered in response to a range of paternal exposures including those described above.^118^ Whilst we are only just beginning to appreciate the implications of such epigenetic changes it is evident that sperm-borne sncRNAs are delivered to the oocyte at the time of fertilisation. Thereafter, sperm sncRNAs have been reported to influence the stability and translational efficiency of maternal transcripts prior to activation of the zygotic genome, and thus alter the trajectory of embryo development and downstream offspring health.^122^, ^123^, ^124^ Whilst the notion that parental experiences exert influence offspring traits independently of the inherited genetic code is not a new concept, until recently these responses have largely been attributed to maternal contributions encountered by the foetus *in utero*.^125^^,^^126^ However, this dogma has been challenged by mounting evidence that environmental exposures can alter ejaculate characteristics, and in particular the sperm sncRNA profile.^17^ Among the first studies to report on this phenomenon, Gapp and colleagues used a pre-clinical mouse model to demonstrate that exposure of male pups to early life trauma not only led to alterations in sperm sncRNA profiles, but also behavioural changes in the offspring of exposed males.^127^ Furthermore, causality was established via recapitulation of equivalent offspring phenotypes after microinjection of total RNA from the sperm of traumatised male mice into fertilised oocytes.^127^ The ensuing decade has borne witness to a rapid growth in the number of studies reporting the ability of various forms of paternal stressors (including diet, psychological stress, alcohol, environmental toxins, cigarette smoke, inflammation, and even exercise), to modify sperm sncRNA profiles leading to readily observable inter/transgenerational phenotypic changes in offspring.^128^ In the subset of studies focusing specifically on the timing and mechanistic basis of these changes, many have converged on the premise that these occur during epididymal transit,^17^^,^^18^^,^^21^^,^^64^ possibly through a common mechanism of dysregulating glucocorticoid receptor expression and signalling in epididymal epithelial cells.^21^^,^^129^ If this mechanism applies broadly, it suggests that somatic cells in the epididymis have the potential to act as environmental sensors, adjusting their physiology in response to diverse paternal exposures and selectively packaging sncRNAs into epididymosomes for delivery to sperm and subsequently the embryo. Importantly, the epididymis provides a protected environment for such modifications, as sperm are transcriptionally and translationally inactive upon entering, making them resistant to direct epigenetic changes. This, in turn, lends support to the notion that the extended period of sperm transit and storage in the epididymis may facilitate the passing of environmental cues to the male gamete.^105^ Whilst we have yet to fully appreciate the connotations of modifying the epigenetic signature of maturing spermatozoa, this phenomenon nevertheless encourages a systematic appraisal of the impact of paternal exposures on the health of future generations.

It is increasingly clear that many of the molecular mechanisms underlying male fertility are directly modulated by lifestyle behaviours. For example, alcohol consumption increases testosterone levels,^62^ impairs Leydig cell morphology,^130^ alters seminiferous tubules^131^ and increases ROS generation in the testes^132^ and epididymis.^133^ Smoking introduces toxicants such as cadmium and polycyclic aromatic hydrocarbons, which induce oxidative stress, lipid peroxidation and reduced sperm motility.^134^^,^^135^ High fat diets can compromise mitochondrial function,^136^ important for the energetic demands of the motility but also the epigenetic content of the fertilising sperm.^18^ Obesity exacerbates hormonal imbalances through increased aromatase activity and oestrogen production, as well as systemic inflammation and disruption of the HPG axis.^137^^,^^138^ These behaviours do not act in isolation but often converge to influence shared biological pathways, including oxidative stress, inflammation, mitochondrial dysfunction, and epigenetic modification, which collectively impair spermatogenesis and reduce sperm quality. Sedentary behaviour further compounds these effects by altering energy metabolism and testicular function.^139^ Understanding how lifestyle factors modulate these molecular mechanisms is essential to guide targeted interventions and reproductive health strategies, particularly in high-risk populations within the WPR.

## Lifestyle behaviour interventions for male preconception health

As outlined above, observational data from cohort studies have demonstrated strong associations between lifestyle and behavioural factors such as diet, physical activity, sleep, weight management, substance use, mental health and male fertility outcomes. In this context, interventions targeting these lifestyle behaviours may be particularly timely and important for male preconception health. This section provides a narrative review of lifestyle behaviour interventions for improving male fertility outcomes across the literature, and where possible a focus within the WPR. Emphasis is placed on evidence derived from high-quality study designs, including systematic reviews, meta-analyses and randomised controlled trials (RCTs), which provide the strongest evidence for intervention effectiveness.

### Dietary interventions

Diet plays a crucial role for male fertility.^46^ While dietary interventions for male reproductive health are limited, a recent systematic review explored the influence of the Mediterranean diet on seminal quality.^52^ The Mediterranean diet is widely studied due to the associated benefits for prevention and management of many chronic diseases.^140^, ^141^, ^142^ It is characterised by high intakes of plant-based foods (e.g., fruit and vegetables) and whole grains, a moderate intake of animal products (e.g., eggs, dairy, poultry), and low intakes of red and processed meat.^141^

The review by Piera-Jordan et al.^52^ identified 10 studies but only two were RCTs. The first RCT^143^ involved 137 men who were residing in Italy and were randomly assigned to a 4-month Mediterranean diet intervention combined with moderate physical activity (The FASt trial). At the end of the program, there was a statistically significant increase in total sperm motility (+3.3%) and progressive motility (+3.8%) in the intervention group when compared with control. However, no significant between-group differences were observed for sperm concentration, volume or morphology. Furthermore, it is unclear whether the independent effects of intervention can be attributed to the Mediterranean diet, given that physical activity was also included as part of the intervention. The second RCT^144^ involved 119 men who were residing in Spain and were randomly assigned to either a Western-style diet enriched with 60 g of nuts per day (nut group) or a Western-style diet avoiding nuts (control group) for 14 weeks. At the end of the program, there were statistically significant improvements in several sperm parameters: total sperm count (mean change: 5.89% versus −21.72%), total sperm motility (mean change: 3.41% versus 0.00%), progressive motility (mean change: 3.78% versus 1.70%), immotile sperm (mean change: −3.41 versus −0.92) and vitality (mean change: 3.42% versus −0.20%) in the intervention group when compared with control. However, no significant between-group differences were observed for sperm concentration, non-progressive motility or normal morphology cells. Overall, the review by Piera-Jordan et al.^52^ concluded that more research is needed to better understand the association between diet and semen quality.

To the best of our knowledge, only one RCT has examined the effects of dietary habits on semen quality within the WPR,^145^ namely Japan, investigating the effects of tomato juice on male infertility. The 12-week trial involved 44 infertile Japanese men and found a significant increase in sperm motility at 6 weeks only (5.87% ± 3.26 versus −3.24% ± 3.35) for the group taking tomato juice (190 g/day containing 30 mg lycopene) compared with the control group (avoiding lycopene-rich foods containing tomatoes). However, this was not sustained at 12 weeks and no significant improvements were observed in any routine sperm parameters.

### Pharmacological and nutrition supplement interventions

Oxidative stress is recognised as key contributor to male infertility.^146^ It occurs when there is an imbalance between reactive oxygen species (ROS) and the body's antioxidant defences,^147^ leading to abnormal sperm parameters and increased sperm DNA fragmentation.^148^ Consequently, the use of antioxidants to mitigate excessive oxidative stress is considered a potential treatment option for male infertility. However, a recent Cochrane Review of 90 RCTs^149^ which explored the effectiveness of antioxidants for male subfertility found the evidence in this area to be '*low*' to '*very low*' quality. Despite these limitations, a meta-analysis was conducted to assess the impact of antioxidant therapy in men on pregnancy and live birth rates. The findings, based on 20 RCTs involving 1706 men, indicated that antioxidants may improve clinical pregnancy rates in partners (OR 1.89, 95% CI: 1.45 to 2.47) compared to placebo or no treatment, with minimal observed heterogeneity (I^2^ = 3%) and overall low-quality evidence. Translating these findings to practical terms, if the baseline clinical pregnancy rate with no treatment is 15%, antioxidant therapy could potentially raise this to between 20% and 30%.

Similarly, antioxidants may lead to increased live birth rates (OR 1.43, 95% CI: 1.07 to 1.91) based on data drawn from 12 RCTs involving 1283 men from very low-quality evidence with observed heterogeneity (I^2^ = 44%). This suggests that if the baseline chance of live birth rate is 16%, antioxidant therapy may raise this to between 17% and 27%. However, this finding was based on just 246 live births from 12 small or medium sized studies. Importantly, when studies at high risk of bias were excluded, there was no significant evidence of increased live birth rate (OR 1.22; 95% CI: 0.85–1.75; p = .27; 8 RCTs; 827 men; I^2^ = 32%). Antioxidant therapy also showed no effect on miscarriage rates.

Amongst the 90 RCTS, 14 were conducted within WPR nations with 9 from China,^150^, ^151^, ^152^, ^153^, ^154^, ^155^, ^156^, ^157^, ^158^ 3 from Japan,^159^, ^160^, ^161^ and 1 each from Australia^162^ and Thailand.^163^ Table 1 summarises the outcomes and conclusions of studies from the WPR, as extracted from the Cochrane review. Notably, 13 of the 14 studies reported a positive effect on male fertility, with most focusing on sperm parameters. While these findings are promising, they should be interpreted with caution due to considerable heterogeneity and high risk of bias.

**Table 1:** Outcomes and conclusions for interventions conducted in the WPR from Cochrane review.^139^

Ref, country, N	Design, population	Outcomes	Results	Conclusions + = Positive effect − = Negative effect
Japan^149^ N = 10	Cross-over, head-to-head Infertile men, high ROS levels	Sperm parameters	Ethylcysteine did not improve sperm density and motility but "sperm function" increased and ROS levels decreased, compared to vitamin E	**+** Ethylcysteine shown to be effective for improvement of sperm parameters when compared to vitamin E
Thailand^153^ N = 68	Multiple arm, placebo, tamoxifen excluded Men with abnormal semen analysis	Sperm parameters, DNA tail length	Folate alone significantly decreased DNA tail length at 3 months. Sperm motility was significantly increased after 3 months Folate alone	**+** Folate in combination with tamoxifen citrate could improve sperm quality including semen parameters and sperm DNA integrity
China^140^ N = 312	Multiple arm, head-to-head Idiopathic OAT	Sperm parameters, DNA fragmentation, pregnancy rate	Significant improvement of sperm parameters and DNA fragmentation in the l-carnitine plus CoQ10 group compared to placebo	**+** Combination of LC and CoQ10 improve semen parameters and outcome of clinical pregnancy
China^141^ N = 86	Head-to-head Men with idiopathic oligoasthenozoospermia	Sperm parameters, adverse reactions, pregnancy rate	Vitamin D is a safe option for the treatment of idiopathic oligoasthenozoospermia and can effectively improve the semen quality especially the progressive sperm motility	**+** Vitamin D can improve forward movement sperm number and percentage, improve the woman's clinical pregnancy rate, and is well tolerated
Japan^150^ N = 75	Multiple arm, no treatment, vardenafil/sildenafil arms excluded Men with oligoasthenospermia	Sperm parameters	An improvement in sperm concentration with carnitine versus no treatment	**+** Enhancement of Leydig cell secretory function may increase sperm concentration and motility
China^142^ N = 769	Parallel, placebo Oligozoospermic men	Sperm parameters, evaluation of MTHFR polymorphism, DNA fragmentation, pregnancy rate, live birth	Folic acid significantly increased sperm parameters decreased oxidative stress and DNA fragmentation and lead to a higher pregnancy and live birth rate in the MTHFR 677 TT group. Effect not seen in other MTHFR groups	**+** Folic acid has a beneficial effect on oligozoospermia with MTHFR 677 TT genotype in terms of sperm parameters, DNA fragmentation and pregnancy outcomes
Japan^151^ N = 396	Multiple arm, placebo Men with abnormal sperm count or motility	Sperm parameters	No statistical difference in sperm outcomes in vitamin B 12 groups or placebo	**−** No improvement in sperm parameters after use of vitamin B12
China^143^ N = 150	Head-to-head Infertile men with OAT	Sperm parameters, pregnancy rate	l-Carnitine and acetyl carnitine more effective than vitamin E + vitamin C for pregnancy, sperm parameters and no evidence of adverse events	**+** l-Carnitine and acetyl carnitine more effective than vitamin E + vitamin C for pregnancy, sperm parameters and no evidence of adverse events
China^144^ N = 80	Head-to-head Infertile men with OAT	Sperm parameters	Statistical significance for carnitines over vitamin E + C	**+** Improvement of sperm parameters for carnitines compared to vitamin E + C
China^145^ N = 54	Parallel, placebo Infertile male patients with varicocele and underwent subinguinal varicocelectomy	Sperm parameters	At 3 months and 6 months after varicocelectomy, the sperm count, the % of motile spermatozoa and the proportions of normally formed spermatozoa in melatonin group were significantly higher than those in placebo group	**+** melatonin therapy adds extra benefit to varicecelectomy in terms of sperm parameters
China^146^ N = 232	Parallel, head-to-head Infertile men with low acrosin activity	Sperm parameters	Significant increase of progressive sperm motility in men treated with l-carnitine compared to vitamin E	**+** l-Carnitine can effectively elevate sperm acrosin activity in male infertility patients, particularly in those with asthenozoospermia
Australia^152^ N = 60	Parallel, placebo Male factor infertility	Pregnancy rate, adverse events, live birth	Antioxidant group recorded a statistically significant improvement in viable pregnancy rate. Side-effects on the Menevit antioxidant were rare (8%) and mild in nature.	**+** Menevit antioxidant appears to be a useful ancillary treatment that significantly improves pregnancy rates in couples undergoing IVF-ICSI treatment. Side-effects on the Menevit antioxidant were rare (8%) and mild in nature
China^147^ N = 135	Head-to-head Infertile men with asthenozoospermia	Sperm parameters, pregnancy rate, adverse events	Significant increase in l-carnitine + vitamin E group for sperm motility, no difference for sperm density and morphology. Pregnancy rate significantly higher in l-carnitine + vitamin E group	**+** l-Carnitine (+vitamin E) significantly improves sperm motility and pregnancy rate
China^148^ N = 120	Parallel, head-to-head Idiopathic asthenozoospermia	Sperm parameters, pregnancy rate	Significant increase of total and progressive sperm motility in vitamin E and vitamin E + compound amino acids group. Greater increase in compound amino acids group. 5.7% pregnancy in combined group, 2% in vitamin E group. No adverse events	**+** Compound amino acid combined with vitamin E can safely and effectively improve sperm motility in idiopathic asthenospermia patients

Note: Adapted from Table 2.^149^

In summary, antioxidant supplementation in sub-fertile males may improve clinical pregnancy outcomes; however, findings are from studies of low quality and from only a few small studies. Variability in findings may be attributable to heterogeneity among studies, including differences in populations, dosages, formulations, treatment duration and outcome measures.^149^ Large well-designed randomised placebo-controlled trials reporting on pregnancy and live births are still required to clarify the exact role and influence of antioxidants.

### Physical activity interventions

There are limited studies examining interventions which focus on improving physical activity levels to enhance male fertility parameters. A recent meta-analysis of six RCTs^164^ found a statistically significant effect of physical activity on sperm concentration, total sperm motility, total sperm count and normal morphology. However, despite these positive findings, the included studies exhibited high heterogeneity, particularly in the duration, type and intensity of physical activity interventions.

To address this variability, a dedicated systematic review and network meta-analysis of seven RCTs^165^ examined the effectiveness of several exercise regimens on male infertility, pregnancy outcomes and seminal markers of inflammation. The different types of exercise included: combined aerobic and resistance training (CET), moderate-intensity continuous training (MICT), resistance training (RT), high-intensity continuous training (HICT), and high-intensity interval training (HIIT). The network meta-analysis found that CET was the most effective intervention across reproductive outcomes (pregnancy rate and semen quality) as well as metabolic health markers (oxidative stress, inflammation, body composition, and VO_2_ max). Notably, MICT improved live birth rates. Therefore, CET and MICT may represent optimal exercise strategies for reproductive and metabolic health in male experiencing infertility.

### Weight loss and weight management interventions

Observational data suggest that maintaining a healthy weight is important for sperm quality.^166^ Despite this, men have largely been overlooked in weight-loss interventions for improving fertility. The most recent meta-analysis on this topic found no lifestyle-based interventions targeting men; all six intervention studies involved either bariatric surgery or medication.^167^ Similarly, a systematic review of 15 randomised controlled trials^168^ reported no eligible trials involving men. Additional reviews, including a meta-analysis of 14 RCTs,^169^ another of eight RCTs^170^ and a narrative review of eight RCTs^171^ also found no studies that included men, once again supporting the gendered imbalance observations of Sharp et al.^13^^,^^14^ A more recent randomised controlled trial^172^ investigated the effects of a low-energy diet (800 kcal/day) in men with obesity, including those with normal sperm concentration (n = 24) or oligozoospermia (n = 43). The 16-week intervention was compared to a control group that received a 10 min session based on national dietary recommendations. Whilst the oligozoospermia patients observed no benefit, obese normozoospermia men exhibited a significant reduction in sperm DNA fragmentation index (−9.5; a marker for oxidative stress).

The systematic reviews and meta-analyses described here did not include any intervention studies conducted in the WPR. To our knowledge, no interventions have been carried out in this region to improve physical activity, or maintained of a healthy weight, for enhancing male fertility parameters.

### Sleep interventions

A systematic review of observational studies has shown sperm parameters to be impacted by short sleep (<5–6 h) and irregular and night work schedules.^173^ However, to our knowledge, there have been no published sleep health promotion interventions that have aimed to improve sleep quality and/or duration to improve fertility outcomes in men.

### Substance use interventions (including alcohol, tobacco smoking and recreational drug use)

Observational studies have shown that substance use including tobacco smoking, alcohol, and recreational drugs have detrimental effects on male fertility.^174^^,^^175^ However, to our knowledge, there have been no published RCTs that have specifically aimed to reduce or eliminate substance use to improve fertility outcomes in men.

### Mental health interventions

Psychological stress has been shown to affect the HPG axis, leading to reduced testosterone and impaired spermatogenesis.^174^ Despite this biological link, there are few mental health interventions specifically designed to help men manage stress and improve fertility outcomes.

While some psychosocial interventions have been conducted with infertile couples, few have focused specifically on men. A recent scoping review^176^ identified 25 psychosocial interventions for infertile couples. However, most of these interventions aimed to improve psychological wellbeing, quality of life, and relationship satisfaction. Only one study focused on improving pregnancy rates through a couple-based intervention.^177^ This study involved 140 Iranian couples experiencing depression. Couples were randomised to either receive six to eight sessions of individual psychotherapy, alongside daily Fluoxetine (20–60 mg), or no intervention (control group), prior to infertility treatment. Results were promising as pregnancy occurred in 47.1% of the intervention group (33 couples), compared to only 7.1% (5 couples) in the control group, with difference between group reaching statistical significance (χ^2^ = 28.318, p < .001).

Systematic reviews in this area have shown that psychosocial interventions could be effective in improving clinical pregnancy rates.^178^^,^^179^ However, as highlighted in the scoping review above,^176^ men are largely absent from these interventions. Across both systematic reviews, only 10 studies examined the effect of psychosocial interventions on pregnancy outcomes, and just two of these included men in RCTs through couple-based interventions. The first of these two studies^180^ included 188 couples who resided in Brazil and were randomised to either intervention group who received 5 weekly 2-h sessions of brief cognitive behavioural therapy (CBT) or no intervention (control group). The pregnancy rate in the intervention group was significantly higher at 39.8%, compared to 23.2% in the control group (χ^2^ = 6.03, p = .01), with an odds ratio of 2.2 (CI: 1.16–4.13), suggesting that participants were more than twice as likely to conceive. In contrast, the second study^181^ involving 122 couples who resided in the Netherlands and were randomised to either an Internet-based intervention including personalised information and communication with clinicians and peers or a control group receiving no additional support. This intervention showed no significant effect on pregnancy rates. The authors suggested that the null findings may be due to the sample size and the fact that the internet-based intervention supplemented rather than replaced usual care, meaning added benefit from the intervention may have been minimal and undetectable in this study.

Although stress is known to negatively affect male fertility, men have been largely overlooked in psychosocial interventions. The few studies involving men, typically as part of couple-based interventions, have shown mixed results when it comes to improving pregnancy outcomes. To our knowledge, there have been no psychosocial interventions implemented within the WPR that have focused on managing psychological distress, depression or anxiety for improving male fertility outcomes. Therefore, further research is critically needed to develop and evaluate culturally relevant interventions addressing these psychological dimensions of male infertility.

## Recommendations

A comprehensive review of the literature on lifestyle behaviours and male fertility has identified several clear recommendations for future research, practice and policy. These recommendations aim to address current gaps and strengthen the evidence base, particularly within the WPR.

### An enhanced molecular understanding of sperm

Greater emphasis is required on utilising multi-omic approaches on sperm such as transcriptomics,^182^ proteomics,^117^^,^^183^ and in particular phosphorylation,^184^^,^^185^ to help decipher molecular mechanisms underlying idiopathic infertility, which accounts for over 70% of cases. Comprehensive profiling of testicular and epididymal contributions to sperm maturation will aid in identifying key markers of fertility potential and subfertility, improving diagnostic precision and tailored treatments.^186^

### Integration of findings into clinical practice

There is a need to integrate these advanced sperm molecular profiling into ART protocols, specifically IVF and ICSI, to enhance fertilisation success rates and improve offspring health outcomes. Clinicians should be trained to interpret and incorporate molecular and epigenetic insights into routine clinical decision-making.

Investigating targeted lifestyle, pharmacological or nutritional interventions aimed at reversing or mitigating epigenetic alterations induced by environmental stressors is also required. These therapeutic options could provide significant benefits to pregnancy outcomes, placental efficiency and improve health and reproductive trajectories for future children.

### Develop, implement and evaluate male preconception health programs

In general, lifestyle behaviour interventions for male preconception health are limited, with greater emphasis on pre-conceptive mothers rather than fathers-to-be. There is a clear need to undertake co-design research with men to better understand their motivators, barriers, and preferences for intervention. These insights can inform the development, implementation and evaluation of accessible male preconception health programs, emphasising lifestyle modifications such as nutrition, exercise, and improved mental health.

Consideration should be given to public awareness campaigns targeting younger populations to underscore the long-term implications of lifestyle choices on reproductive health, even among those not currently planning parenthood. School-based education programs could effectively integrate such content, normalising early preventive behaviours.

### Expand research in the Western Pacific Region

The evidence base for the impact of lifestyle behaviours on male fertility have predominately been conducted outside the WPR. As a result, more research is needed within the WPR to account for differences in key determinants of health, including cultural, lifestyle, environmental, societal, economic, and healthcare system factors.

### Greater focus on understudied lifestyle behaviours (i.e., substance use, diet, sleep, weight loss, mental health)

Beyond pharmacological and nutrition supplement interventions there is a dearth of evidence on lifestyle behaviours interventions targeting male preconception health. This suggests a lack of accessible resources and programs to support sustainable lifestyle changes. There is a clear need for more high-quality RCT's to assess the effectiveness of other lifestyle behaviours (e.g., substance use, diet, sleep, weight loss, mental health) to better understand their influence on male preconception health. Such evidence can inform development of resources and programs to support men in making positive lifestyle changes but also equip health professionals with clear guidance on effective recommendations.

### Ensure methodological rigour and standardisation

Finally, many of the intervention studies were limited by small sample sizes, short follow-up periods and heterogeneity. To enhance overall quality of evidence, there is a need for more large-scale, long-term RCTs with standardised clinically meaningful measures to enable comparison across studies. Many studies have evaluated the impact of lifestyle interventions on sperm parameters such as count, motility and morphology. While these are important indicators of male fertility, improvements in sperm parameters do not always translate to increase pregnancy or live birth rates.^187^ There is a need for more studies that evaluate clinically meaningful outcomes such as clinical pregnancies and live birth rates in addition to sperm parameters.

## Policy implications—prioritisation and funding

Policymakers need to prioritise male reproductive health by increasing research funding, developing infrastructure for better male-focused fertility services, and encouraging cross-disciplinary collaborations. Additionally, regional policies should ensure equitable access to ART services, especially in underserved areas of the WPR.

Efforts should focus on strengthening regional policy frameworks to mitigate exposure to endocrine-disrupting chemicals, pollutants, and occupational hazards affecting male fertility. Harmonised and stringent environmental regulations should be advocated alongside improved workplace safety standards to protect reproductive health.

## Declaration of generative AI and AI-assisted technologies in the writing process

During the preparation of this work the authors used OpenAI (2025), ChatGPT (May 2025 version GPT-4) in order to ensure readability and clarity of the text. After using this tool, all authors have reviewed and edited the content as needed and collectively take full responsibility for the content of the publication.

## Contributors

CRediT Author statement contributions: All authors contributed to: conceptualisation, methodology, writing—original draft, iInvestigation, and reading and approving the final manuscript. Additionally, D.S.B. contributed to visualisation (compilation of figures) and project administration, L.M.A. contributed to project administration and both B.N. and P.J.M. contributed to supervision.

## Data sharing statement

This review is a summary of peer-reviewed articles which are included in the reference list and readers may access as required.

## Declaration of interests

The authors declare that they have no known competing financial interests or personal relationships that could have appeared to influence the work reported in this paper.
